# Effect of Feed Restriction and Realimentation with Monensin Supplementation on Placental Structure and Ultrastructure in Anglo-Nubian Goats

**DOI:** 10.5402/2012/490530

**Published:** 2012-09-10

**Authors:** A. L. Cristofolini, M. P. Turiello, E. G. Sanchis, G. Cufré, C. I. Merkis

**Affiliations:** ^1^Área de Microscopía Electrónica, Facultad de Agronomía y Veterinaria, Universidad Nacional de Río Cuarto, Ruta Nac, 36-Km, 601, Córdoba, X5804BYA Río Cuarto, Argentina; ^2^Cátedra de Nutrición Animal, Área de Microscopía Electrónica, Facultad de Agronomía y Veterinaria, Universidad Nacional de Río Cuarto, Ruta Nac, 36-Km, 601, Córdoba, X5804BYA Río Cuarto, Argentina; ^3^Becaria CONICET/MinCyTCba, Área de Microscopía Electrónica, Facultad de Agronomía y Veterinaria, Universidad Nacional de Río Cuarto, Ruta Nac, 36-Km, 601, Córdoba, X5804BYA Río Cuarto, Argentina

## Abstract

The aim of this study was to evaluate the effect of feed restriction followed by a realimentation with monensin supplementation on morphological, ultrastructural, and apoptotic characteristics in the term placenta of Anglo-Nubian does. Treatments were a control group (C = 5), a group fed at 0.70 of that consumed by controls (R = 7), and the same as R with monensin (M = 7). After parturition, 27 placentas were gathered, C: 7, M: 10, and R: 10. No differences were detected between treatments in relation to morphological and ultrastructural analysis. The greatest values of binucleate cells were detected in placentas from R, and it could be due to the need to compensate and satisfy nutritional differences of restriction. We detected the highest apoptotic index in R as a consequence of nutritional treatment. We describe for the first time the structural and ultrastructural morphology and remodeling by apoptosis of Anglo-Nubian placenta at term of goats subjected to nutritional restriction during peripubertal period and the use of monensin as a growth promoter.

## 1. Introduction

Caprine placenta is classified as cotyledonary and characterized by the development of restricted areas of interdigitation between fetal and maternal tissues known as placentomes [1]. This placenta has been also classified as synepitheliochorial due to the occurrence of migration of fetal chorionic binucleate cells across the microvillar junction to fuse with the maternal epithelial cells and to form the syncytium [2]. 

As other ruminants, the goat has a characteristic placental epithelium with two morphologically and functionally distinct cell types in the trophectoderm that are the mononucleate and the binucleate trophoblast cells [3]. Mononucleate cells are the most commonly found in the interface and are primarily involved in the nutrient exchange. Binucleate cells produce hormones, such as placental lactogen and progesterone [3], and through the fusion process with an uterine epithelial cell or with the fetomaternal syncytium release their content to the maternal connective tissue [4]. Binucleate cells are also involved in villi development [4] and are a unique feature in ruminants [5]. In caprines, their role in maturation and delivery of placenta is yet unknown [6]. 

The placenta is designed to allow an efficient exchange between mother and fetus to optimize the growth and development [7]. Furthermore, a correlation between placental and litter weight has been determined [8, 9]. In turn, the term placental efficiency has been considered as an indicator of uterine capacity, suggesting that smaller placentas are more efficient since they would need fewer nutrients. In addition, the number of placentomes decreases under extreme conditions such as heat stress or malnutrition of newborn [8].

Programmed cell death constitutes an essential factor in placental development [10] and together with cell proliferation plays an important role, both processes being inversely proportional: as proliferation increases, apoptosis decreases [11]. It has been demonstrated that apoptosis increases as pregnancy progresses in humans, suggesting it plays an important role in trophoblastic villi differentiation and syncytium formation [12]. At the beginning of swine gestation, apoptosis has been related with placental villi homeostasis through cell remodelling [10, 13–15], while at the end of humans and swines pregnancy, cell remodelling by apoptosis would be contributing to fetal and maternal membranes separation [13, 16]. Apoptotic index could be modified under stress conditions. A high level of apoptosis in trophoblast has been associated with fetal membrane retention in cattle [11] and with preeclampsia and fetal growth retardation in humans [17]. There is no evidence of apoptosis in term goat placenta.

Reproduction is affected by nutrition [18]. Severe undernutrition prevents ovulation in lambs by impairing the system governing GnRH secretion [19] and, consequently, LH secretion [18, 20]. Cufré et al. [21] demonstrated that puberty onset was limited with a feed restriction of 30% in Anglo-Nubian does but with a moderate restriction of 20% puberty was not inhibited. However, we have demonstrated that puberty may be achieved in the second breeding season when they are refed (unpublished data).

To promote feed use efficiency, ruminal fermentation may be manipulated with the use of ionophores as monensin [6]. Ionophores improve energy metabolism efficiency in ruminants promoting propionate production and reducing methane synthesis [22]. For growing goats more information is needed about monensin effectiveness over growth [23]. There is no evidence in relation to term placental traits of goats previously subjected to feed restriction and realimentation with long-term monensin supplementation. 

The description of the apoptotic process in placenta of goats subjected to different nutritional planes would contribute to the knowledge of the mechanism of programmed cell death and its influence on placental development under both physiological and stress conditions.

The aim of this study was to evaluate the effect of feed restriction followed by a realimentation with monensin supplementation on morphological, ultrastructural, and apoptotic characteristics in term placenta of Anglo-Nubian does.

## 2. Materials and Methods

### 2.1. Animals and Treatments

The experiment was carried out during 21 months at the facilities of the Universidad Nacional de Río Cuarto, Córdoba, Argentina, 33° 08′ S, 64° 20′ O′. 19 prepubertal Anglo-Nubian does (BW = 11.9 ± 1.47, *P* = 0.991) from a local herd were randomly allocated to one of three treatments, based on feeding conditions. Treatments were a control group, fed to appetite (C; *n* = 5); a group fed at 0.70 of that consumed by controls adjusted to a common BW basis (R; *n* = 7); the same as R but supplemented with monensin (M; *n* = 7). The level of dry matter feed intake for R and M during restricted period was adjusted using the following formula: [0.7 × (BW_R,M_/BW_C_) × DMI_C_], where BW_R,M_ is the mean body weight (BW) of R or M, BW_C_ is the mean BW of C, and DMI_C_ is the mean daily dry matter intake of C. Does were restricted for 250 days, and after this period they were refed for the rest of the trial. In both periods, diet had 2.3 Mcal ME/kg DM and consisted of 0.70 chopped alfalfa hay supplemented with 0.30 of ground maize grain. All animals received a daily supplement of 7 g of a vitamin-mineral mixture, and animals from treatment M were supplemented with 12.5 mg monensin (as sodium monensin, 0.20). Does were daily placed in individual cages for 8 h to allow individual feeding, and the rest of the day were housed in groups into 8 × 4 m common pens, with free access to fresh water, allowing for social interaction. After 160 days of refeeding, does were mated with a fertility-proved male, and they all got pregnant.

### 2.2. Placental Collection and Macroscopic Analysis

Immediately after parturition, 27 placentas were gathered (C: 7, M: 10; R: 10), washed with Hank's saline solution (SSH) (Gibco, USA) containing 10,000 U/mL of penicillin G sodium, 10 mg/mL of sulfate of streptomycin, and 2.5 *μ*g/mL of fungizone (Gibco, USA) and then maintained at 4°C up to their processing in the laboratory. The placentas were weighed, the placentomes were counted, and the placental efficiency was calculated as the relation between the average weight of the fetus and the average weight of the placenta from each doe. The cotyledon density was calculated as the number of cotyledons in gram of placental weight. Samples from 5 cotyledons per placenta were taken and placed in buffered formaldehyde for optical microscopy analysis. Samples from 5 cotyledons per placenta were placed in glutaraldehyde for electron microscopy analysis. The cotyledons were selected from placental zone itself.

### 2.3. Morphological Analysis

#### 2.3.1. Light Microscopy

Samples were processed by conventional histological technique. Portions of placental tissue of approximately 6 mm^3^ each were fixed by saline-buffered formaldehyde and dehydrated by increasing graduation alcohol battery to be included in paraffin. Histological samples of 4 *μ*m thick were stained by haemotoxylin-eosin and Masson's trichrome dyes for structure recognition and analyzed at light microscopy.

#### 2.3.2. Electron Microscopy

Samples of placental tissue of 2 × 2 mm were fixed with 2.5% (v/v) glutaraldehyde in buffer s-collidine pH 7.2–7.4, 2 h at room temperature and washed with s-collidine. They were refixed 1 h with 1 : 1 osmium tetroxide-S-collidine, washed with s-collidine, and dehydrated with increasing graduation acetone battery. Samples were placed in EMbed-812 resin in 100% acetone (1 : 1) for 24 h to preinclusion and then in EMbed 812 with a catalyst at 60°C for 24 h to final inclusion. Ultrathin sections (±600 Å) were obtained using an ultramicrotome (Sorvall MT-1A, DuPont, USA) and a diamond cleaver. Then, sections were mounted on copper grids, double-stained with uranyl acetate and lead citrate as usual, and examined with a transmission electron microscope Elmiskop 101 (Siemens, Germany).

#### 2.3.3. High Resolution Light Microscopy

For high resolution light microscopy (HRLM) the semithin sections (±0.25 *μ*m) obtained for transmission electron microscopy technique were used. The semithin sections were counterstained with toluidine blue and were cover-slipped in DPX (Merk, Alemania) embedding agent. The sections were observed in a light microscope Axiophot (Carl Zeiss, Thornwodd, NY) fitted with a high-resolution digital camera Powershot G6 7.1 megapixels (Canon INC, Japan). Digital images were captured with Axiovision Release 4.6.3 software (Carl Zeiss, Göttingen, Germany). The results were expressed as quantitative. From the relation between the number of binucleate cells and the number of total cells, the binucleate cell index (Ibc) for the different groups was determined, using the following formula:

(1) $$\begin{array}{c} \text{lbc}=\frac{\text{Binucleate cells}}{\text{Total cells}}\times 100. \end{array}$$

### 2.4. TUNEL Technique, Quantification of Apoptotic Index, and Statistical Analysis

Nuclei DNA fragmentation was detected *in situ *using the terminal- deoxynucleotidyl- transferase- (TdT-) mediated dUTP nick end labelling (TUNEL) method (ApopTag Plus Peroxidase In Situ Apoptosis, Chemicon International, USA) [24]. Analysis was conducted as described in kit protocol, following the recommendation of a pretreatment of slides with tritium. The results were expressed as quantitative. From the relation between the number of cells with fragmentation of DNA and the number of total cells, the apoptotic index (IAp) for the different groups was determined, using the following formula:

(2) $$\begin{array}{c} \text{lAp }=\;\frac{\text{TUNEL}\;-\text{ positive cells}\;}{\text{Total cells}}\;\;\times \;\;100. \end{array}$$

### 2.5. Statistical Analysis

Results were analyzed statistically by ANOVA (InfoStat) [25], according to a completely randomized design. Placental traits (weight, number of placentomes, and apoptotic index) were analyzed according to the following statistical model: *Y*_*ij*_ = *μ* + *D*_*i*_ + *e*_*ij*_, where *Y*_*ij*_ is the variable under consideration, *μ* is the overall mean, *D*_*i*_ is the fixed effect of dietary treatment, and *e*_*ij*_ is the associated error. In those cases when treatment differences were detected, means were compared by the least significant difference at a *P* < 0.05.

## 3. Results

Of 19 births, 12 were multiple (11 doubles and 1 triple) and 7 simple ones. All the placentas were expelled in a period of 3 hours after parturition. Mean placental weight was 457 ± 140.6 g, and no differences were detected between groups for this variable neither for cotyledon number nor for placental efficiency (Table 1). The results of this study show positive correlations between cotyledon number (CN), placental efficiency (PE), cotyledon density (CD), and litter weight (LW) (*r* = 0.6, 0.53, and 0.58). Furthermore, cotyledon density was positively correlated with cotyledon number and placental efficiency (*r* = 0.57, and 0.70). A negative correlation between placental weight and placental efficiency and cotyledon density was found (*r* = −0.67 and −0.55 (Table 2)).

**Figure 1:**
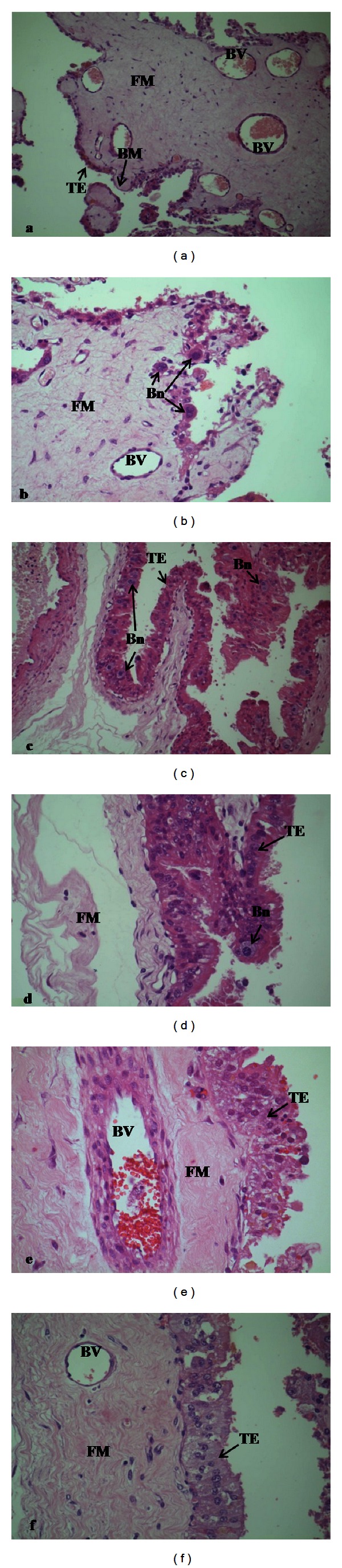
Photographies of at term goat placenta stained with haematoxylin-eosin. (a) and (b) C-group, (c) and (d) M-group, (e), and (f) R-group ((a) 100x; (b), and (c) 200x, (d), (e) and (f) 400x). BM: basal membrane, Bn: binucleate cells, BVs: blood vessels, FM: fetal mesenchyme, TE: trophoblastic epithelium.

In the trophoblastic epithelium, cylindrical cells arranged in palisade and numerous binucleate cells can be visualized. In addition, it can be appreciated as fetal mesenchyme with elongated cores, for being a connective lax tissue, very vascularized with blood vessels of diverse caliber, some located very near the epithelium (Figure 1).

**Figure 2:**
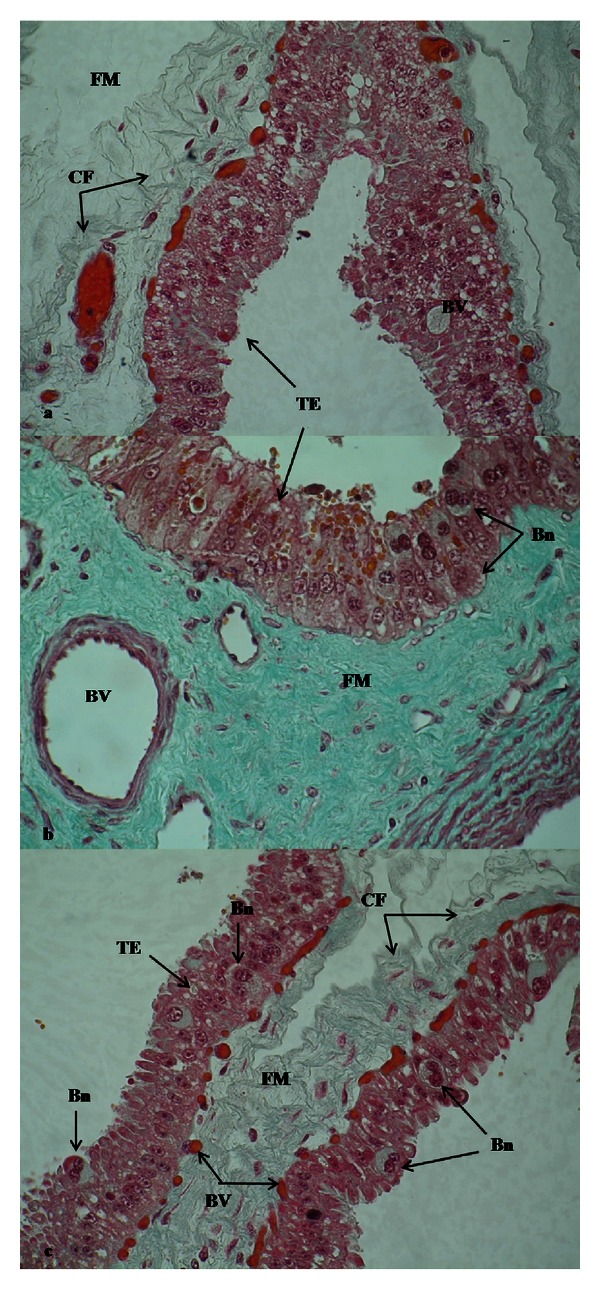
Placental sections from R-group (a), M-group (b), and C-group (c), stained by Masson's trichrome dyes (400x). Bn: binucleate cells, BVs: blood vessels, CF: collagenous fibers, FM: fetal mesenchyme, TE: trophoblastic epithelium.

In placental sections from R-group, M-group and C-group, through Masson's trichrome technique placental sections from groups R, M and C can be seen with more precision, specifically cells disposed in palisade and fetal mesenchyme constituted by connective lax tissue with collagenous fibers. In addition, it shows medium and small blood vessels near the basal membrane (Figures 2(a), 2(b), and 2(c), resp.).

**Figure 3:**
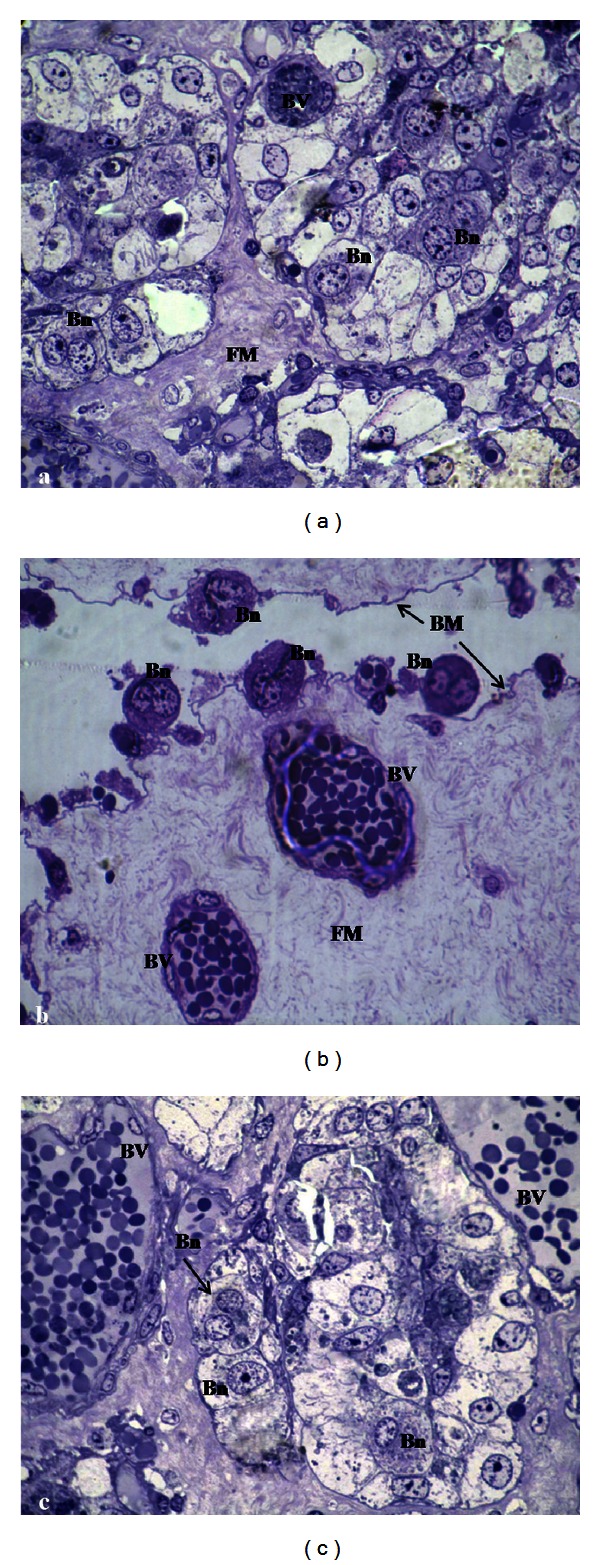
HRLM of goat placenta from R-group (a), M-group (b), and C-group (c) (1000x). BM: basal membrane, Bn: binucleate cells, BVs: blood vessels, FM: fetal mesenchyme.

Through the placental structural analysis by HRLM (Figures 3(a), 3(b), and 3(c)) the Ibc of each group ranged between 15% and 17%, reaching the maximum values in R-group. 

No differences were detected between treatments in relation to morphological analysis.

**Figure 4:**
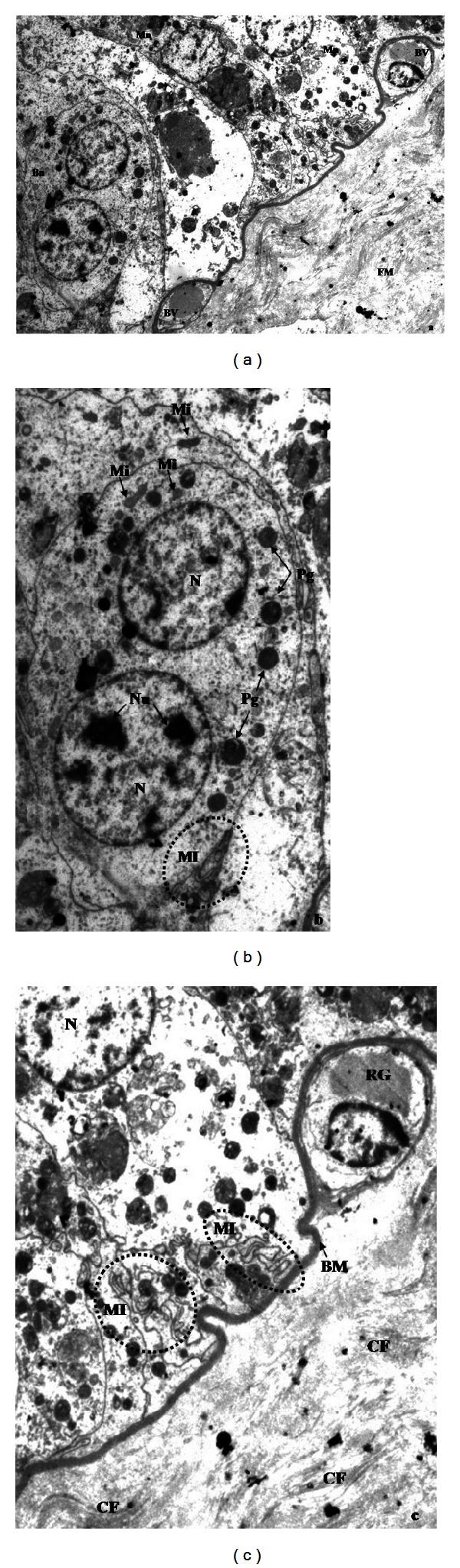
Photomicrography of goat placenta from R-group ((a) 2156x; (b) and (c) 3597x). BM: basal membrane, Bn: binucleate cell,BVs: blood vessels, CF: collagenous fibers, FM: fetal mesenchyme, Mi: mitochondria, MI: membrane interdigitations, Mn: mononucleate cell, N: nucleus, Nu: nucleolus, Pg: particle containing granules, RG: red globule.

In R-group placentas analysed by TEM, trophoblastic epithelial cells arranged in palisade and settled on a very clear basal membrane were observed. In addition, mononucleate epithelial cells metabolically active were seen, characterized for being cylindrical, with central, rounded cores, rich in euchromatin. Numerous cytoplasmic organelles, as mitochondria and granules-containing particles, could also be distinguished. Intercellular communication is outlined between adjacent epithelial cells across membrane intervilli. Binucleate cells have ultrastructural characteristics similar to mononucleate cells. Our observations corroborate the closeness of some small blood vessels to the trophoblastic epithelium. In addition, collagenous fibers of different thickness are immersed in the fetal mesenchyme (Figure 4).

**Figure 5:**
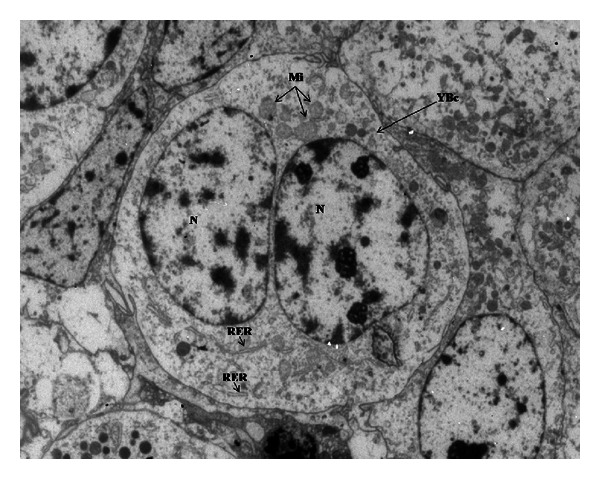
Photomicrography of young binucleate cell in goat placenta from R-group (3697x). N: nucleus, Mi: mitochondria, RER: rough endoplasmic reticulum, YBc: young binucleate cell.

**Figure 6:**
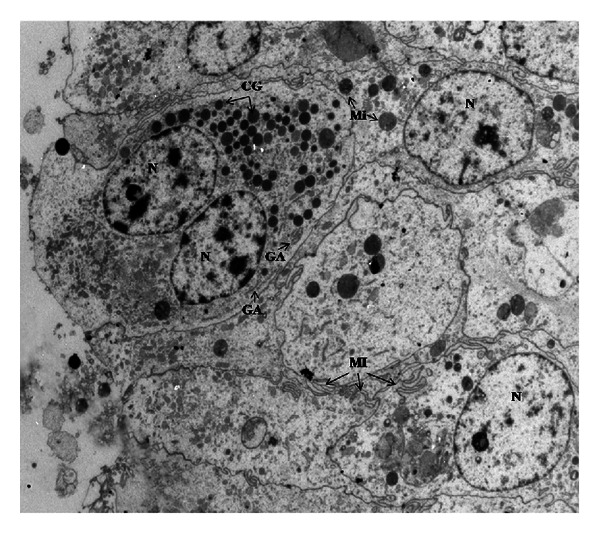
Microphotography of mature binucleate cell in goat placenta from R-group (2784x). CG: cytoplasmatic granules, GA: Golgi apparatus, Mi: mitochondria, MI: membrane interdigitations, N: nucleus.

**Figure 7:**
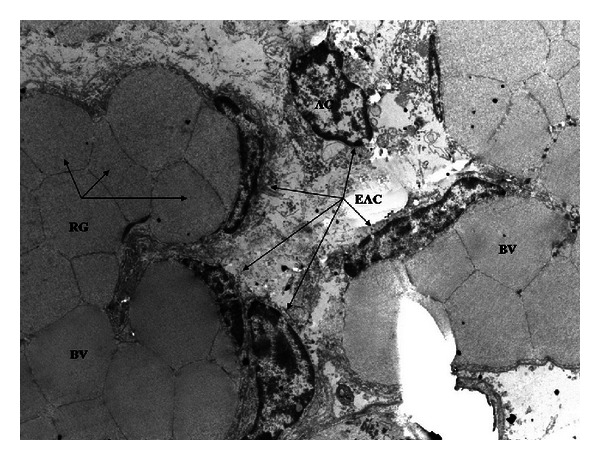
Microphotography of goat placenta from M-group (3597x). AC: apoptotic cell, BV: blood vessels, EAC: endothelial apoptotic cell, RG: red globule.

In R-group placenta young binucleate cell (YBc) and mature binucleate cell (MBc) were determined. Young binucleate cells have fewer mitochondria, a dense cytoplasm with numerous ribosomes and no glycogen (Figure 5). Ultrastructure of mature binucleate cells is characterized by numerous cytoplasmatic granules and large Golgi bodies (Figure 6). In fetal mesenchyme of M-group placenta the presence of big blood vessels, containing numerous red blood granules, was observed. Some endothelial cells have ultrastructural apoptotic characteristics, such as the presence of electrodense nuclear material because of heterochromatin condensation, reduction of cellular size, and integrity maintenance of plasmatic membrane. The presence of cells in apoptosis in connective tissue was also observed (Figure 7).

**Figure 8:**
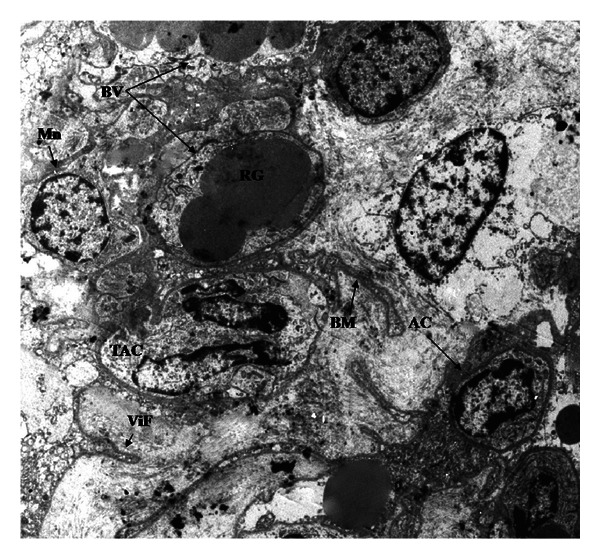
Photomicrography of goat placenta from R-group (2784x). AC: apoptotic cell, BM: basal membrane, BV: blood vessel, Mn: mononucleate cell, TAC: trinucleate apoptotic cell, RG: red globule, ViF: trophoblastic villi fold.

We highlight the finding through the ultrastructural analysis of R-group placenta of an outstanding trinucleate cell in placental trophoblastic villi. It showed evidence of programmed cell death with increased cytoplasmic density and decreased cellular size (Figure 8).

Some mononucleate cells have also morphologic characteristics of an apoptotic process in different states of development. The presence of numerous blood vessels located very near the trophoblastic epithelium was very clear. 

There were no differences in ultrastructural characteristics between treatments.

By TUNEL technique, apoptotic cells could be identified through visualization of nuclei with chromatinic marginalization. TUNEL-positive nuclei showed a random distribution in trophoblastic placental villi.

**Figure 9:**
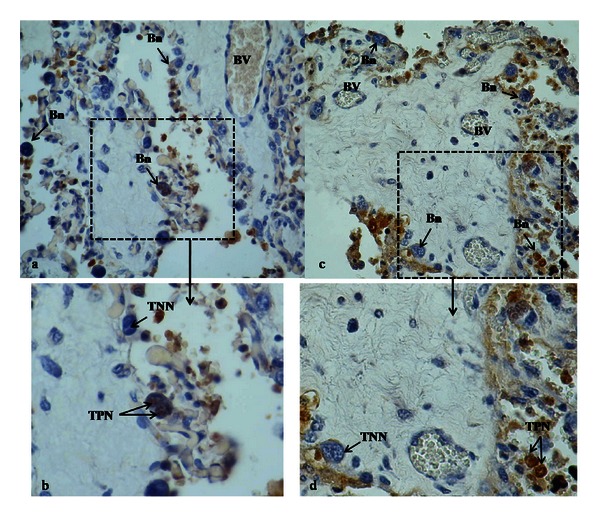
Photographies of TUNEL technique in goat placenta at term gestation where apoptotic nuclei were observed. (a) and (b) R-group; (c) and (d) M-group ((a) and (c) 400x; (b) and (d) 1000x). Bn: binucleate cell, BVs: blood vessels, TNN: TUNEL negative nuclei, TPN: TUNEL positive nuclei.

In Figures 9(b) and 9(c) TUNEL-positive binucleate cells can be seen although almost all of them were metabolically active, rounded in shape, with two nuclei and a more intensive staining. The apoptotic index (IAp) based on TUNEL labelling showed differences between R-group and the other groups (Table 3). No differences were detected between C-group and M-group.

## 4. Discussion

Gestation lasted in average 147.9 days, 3.2 days less than cited by Amoah et al. [26] in Anglo-Nubian goats and 1.3 days less than determined by Dickson-Urdaneta et al. [27] for Nubian goats. In caprines, the duration of gestation raises together with the increase of the number of births; however, in this study, we worked with first birth does [26]. Significative differences were found among groups, with 2.6 days in favour of control group compared to restricted groups. There were no statistical differences among groups according to litter size and sex that could justify the differences in the duration of gestation among groups. Nevertheless, the frequency of multiple births was higher for does from M and R groups against those from C-group, which could explain the difference in the lasting of gestation.

There were no differences among groups according to delivery rate and litter weight. M-group showed the highest value in the frequency of multiple births, followed by R-group and, finally, by C-group. Mellado et al. [28] determined that the nutrition of does during early growth constitutes a determining factor for litter size, since it affects the ovulation rate; however in the present work there was no effect of nutritional restriction during peripubertal period on the size of the litter. 

Values of prolificity determined in this study varied between 1.4 and 2.1 kid/goat, in coincidence with the previously informed in Nubian goats [27, 29]. Moreover, average weight at birth varied between 3.1 and 3.3 k, as has been previously stated [8, 26]. 

In the present study placentas from simple and multiple gestations were obtained. No significant differences were detected among groups. Additionally, there were not statistical differences among groups in placental weight, number of cotyledons, and morphological analysis, indicating that previous treatment did not affect placental development and morphophysiological characteristics. Nevertheless, goats from R-group showed a diminution of placental size and an increase in number of cotyledons, causing an augmentation of placental efficiency, since larger placentas require a higher demand of nutrients [8, 30].

Through structural analysis, we have corroborated the described characteristics of goat placentas, such as the separation of maternal and fetal tissues at trophoblast level. At term placenta does not present maternal tissue, together with the loss of fetal epithelium in some villi areas [31]. Additionally, viable binucleate cells, typical from ruminant placenta, were found immersed among cell debris due to the rupture during birth of the trophoblastic epithelium at the level of fetal mononucleate cells [5]. 

Young and mature binucleate cells from trophoblast were ultrastructurally differentiated by means of the use of transmission electron microscopy, showing the presence of cytoplasmic granules containing placental lactogen and progesterone, proteins that transfer through the fetoplacental barrier and are involved in the development of cotyledonary villi system [3–5, 31].

Number of trophoectodermal binucleate cells was determined by means of high-resolution light microscopy, and our data is coincident with that described by Wooding and Burton [31] in gestations of 135–148 days. However, the greatest values of binucleate cells detected in placentas from R-group could be due to the need to compensate and satisfy nutritional differences. 

Effect of treatment was detected over the apoptotic characteristics of the studied placentas. TUNEL assay has been used in other placentation types as an undisputed tool for determination of DNA fragmentation in initial stages of the apoptotic process [32–34]. Particularly, in pigs, TUNEL has allowed the detection of apoptotic levels, so early as at 28 days of gestation [10]. In the present study, the differences observed in apoptotic index (IAp) of R-group reflect the effect of nutritional restriction during peripubertal period of goats. We detected the highest apoptotic index in goats subjected to nutritional restriction without supplementation (R-group), presumably as a consequence of nutritional treatment; others have described an increase in the number of apoptotic cells to the end of gestation due to the normal development of the placenta [11]. In this way peripubertal nutritional restriction could be associated with a diminution of the viability of cells from placental villi. 

It is important to highlight that goats from M-group did not show differences with goats from C-group, despite being subjected to the same restriction level of those of R-group. Monensin regulates numerous cell functions, including apoptosis, and has an *in vitro* antiproliferative effect on tumor cell lines inducing that type of cell death [35]. However, in the present work and at the dose used, addition of monensin to the diet had no adverse effect over the placental remodeling by apoptosis in caprine placenta. 

As observed in goats from M-group, it has been determined that monensin supplemented prepartum dairy cows improved their energy balance, preventing some health problems, such as retention of fetal membranes [36]. With the present results we confirmed an effect of the ionophorus on growth and maternal body condition of goats that lead to normal placental cell remodeling and development. 

The presence of ionophorus residues has been demonstrated in bird eggs and tissues [37, 38]; however it has been proved that there is no accumulation in bovine tissues after its oral administration [39]. No information is available regarding the effect of monensin on ruminant placentas; therefore, the knowledge of *in situ* effects of monensin on caprine placenta would provide an interesting perspective for future studies. 

In this work, we described for the first time structural and ultrastructural morphology of at term Anglo-Nubian placenta of goats subjected to nutritional restriction during peripubertal period and the use of monensin as a growth promoter. This is, as well, the first finding of placental cell remodeling by apoptosis in at term caprine placenta. 

To deepen the knowledge of cell remodeling through caprine gestation and the determination of the pathways involved in the apoptotic process during feed restriction will allow to generate different strategies applicable to livestock production in the future in order to improve the activity in this high productive value species.

## Figures and Tables

**Table 1:** Mean placental weight (PW), cotyledon number (CN), cotyledon density (CD), and placental efficiency for C, M, and R.

	Treatment (mean ± SE)*			SD
	RowSpanEmpty	C	M		R
PW (g)	539.4 ± 143.13	437.6 ± 166.31	417.9 ± 100.50	138.855
CN	86.6 ± 26.04	87.0 ± 24.43	94.8 ± 27.85	26.041
CD (g^−1^)	0.17 ± 0.07	0.22 ± 0.10	0.20 ± 0.11	0.100
PE	6.5 ± 2.33	6.5 ± 1.92	8.4 ± 1.91	2.000

SD: square root of the CM of the error of each ANAVA.

*NS (*P* > 0.05).

**Table 2:** Correlation coefficients (*r*) between litter weight (LW), cotyledon number (CN), placental efficiency (PE), cotyledon density (CD), and placental weight (PW).

	CN	PE	CD (g^−1^)	PW (g)
LW (g)	0.60*	0.53*	0.58*	0.25
CN		−0.02	0.57*	0.05
PE			0.70*	−0.67*
CD (g^−1^)				−0.55*

*P < 0.05.

**Table 3:** Apoptotic index in placentas from C, M, and R treatments.

Treatment	IAp*
C	0.23 ± 0.01^a^
M	0.21 ± 0.02^a^
R	0.28 ± 0.02^b^

*P < 0.0001.
